# Improving genetic transformation rates in honeybees

**DOI:** 10.1038/s41598-018-34724-w

**Published:** 2018-11-08

**Authors:** M. Otte, O. Netschitailo, O. Kaftanoglu, Y. Wang, R. E. Page Jr., M. Beye

**Affiliations:** 1 Evolutionary Genetics, Heinrich Heine University Düsseldorf, Düsseldorf, Germany; 2 School of Life Sciences, Arizona State University, Tempe, United States; 3 Department of Entomology and Nematology, University of California, Davis, United States

## Abstract

Functional genetic studies in honeybees have been limited by transformation tools that lead to a high rate of transposon integration into the germline of the queens. A high transformation rate is required to reduce screening efforts because each treated queen needs to be maintained in a separate honeybee colony. Here, we report on further improvement of the transformation rate in honeybees by using a combination of different procedures. We employed a hyperactive transposase protein (hyPBase^apis^), we tripled the amount of injected transposase mRNAs and we injected embryos into the first third (anterior part) of the embryo. These three improvements together doubled the transformation rate from 19% to 44%. We propose that the hyperactive transposase (hyPBase^apis^) and the other steps used may also help to improve the transformation rates in other species in which screening and crossing procedures are laborious.

## Introduction

Social insects such as the honeybee display interesting behaviours and developmental processes. A honeybee colony typically consists of thousands of worker bees, a single queen and hundreds of males (drones). The worker bee caste displays a rich repertoire of altruistic behaviours^1–5^, sophisticated cognitive abilities^6,7^, and communication abilities^8,9^. Moreover, the many behavioural activities together collectively contribute to colony growth and maintenance^10–15^. The queen caste displays egg-laying behaviours within the colony, while the drones devote their behaviours to mating. The development of morphologically distinct queens and workers is controlled by the combination of females with the caste-determining pathway^16^. The female determination signal is provided by a heterozygous genotype at the *complementary sex determiner* (*csd*) gene (two different sex-determining alleles)^17,18^. Female larvae develop into either a queen or a worker bee depending on the food that the female larvae receive from the worker bees^19–22^. A systematic dissection of the molecular underpinnings of the honeybee using transgenic systems is still limited. The usual low transformation rates limit the application of genetic transformation systems because, in the search for a transgenic queen, each treated and reared queen (the only reproductive female) needs to be maintained in a separate colony. In a previous study, we demonstrated that we can transform queens with the *piggyBac* transposon^23^. However, when we increased the size of the transposon cassette (consisting of pBacL, pBacR, promotor and gene of interest) from 2.5 to 5.2 kb, we detected a low number of transgenic offspring among the many queens that we screened^23^, suggesting that we need to further improve the transformation rates. Yusa *et al*. reported a hyperactive variant of the *piggyBac* transposase (hyPBase)^24^ using mutational screening experiments, they identified seven amino acid changes that enhanced *piggyBac* transposition in mice by 10-fold compared to a codon adjusted transposase of mice. Codon usage adjustments were shown to affect the transformation rate; when codon usage of the co-transfected transposase gene was adjusted to that of the mice, the transposition rate increased by up to 20-fold in mouse embryonic stem cells^25^. Furthermore, the area of injection into the embryo is a critical parameter for a high transformation rate. In the silkworm *Bombyx mori*, Tamura and co-workers showed that changes in the injection position and direction (possibly by improving the targeting of the germ cells) tripled the number of transgenic silkworms obtained^26^.

Here, we report a twofold increase in transformation rates in honeybees by employing a hyperactive transposase with honeybee codon usage, by increasing the amount of injected transposase mRNAs, and by injecting into the anterior part of the embryo in order to target the first nuclei. The combination of these optimized procedures may also be applied to other insect species to increase transformation rates.

## Results

**Figure 1:**
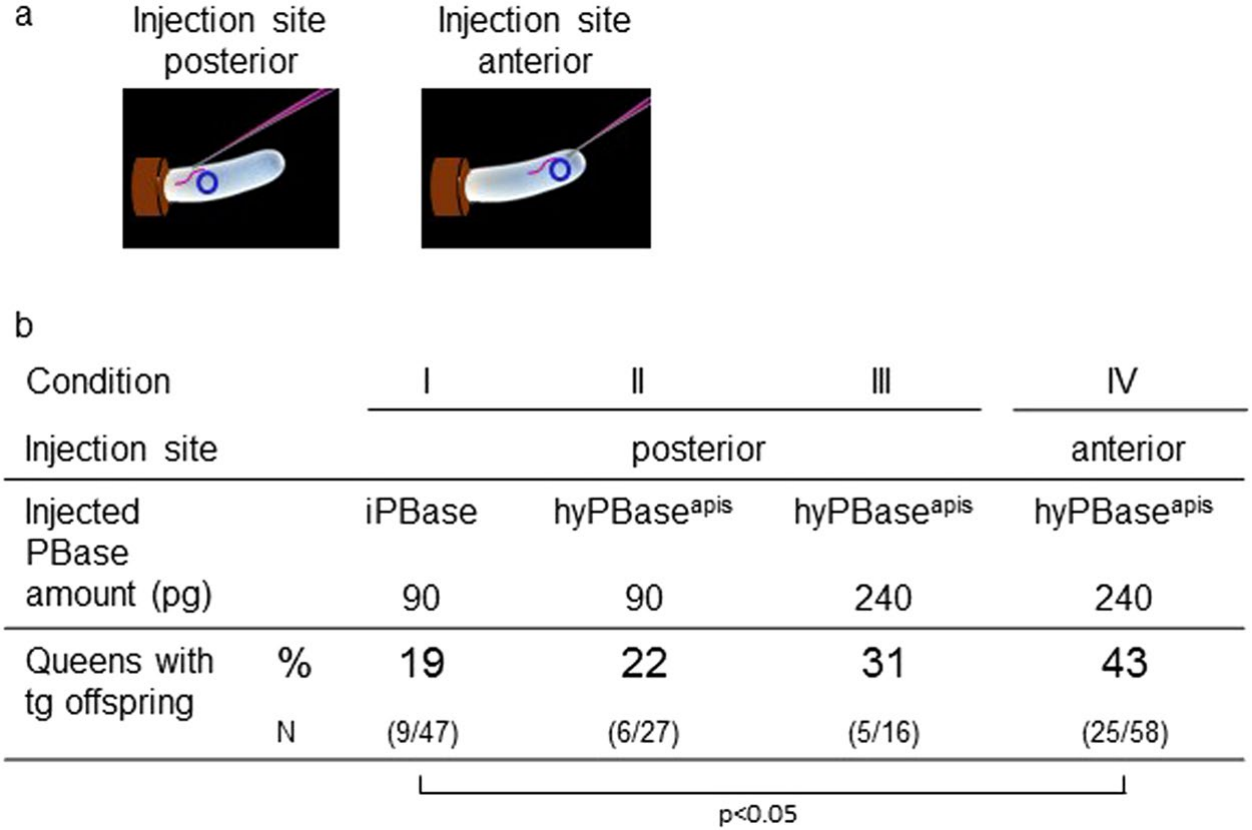
Transformation rates of honeybee queens using different transposases (iPBase and hyPBase^apis^), transposase mRNA concentrations and injection sites. Honeybee embryos were injected at either the posterior or the anterior site (**a**) with 90 pg or 240 pg of iPBase and hyPBase^apis^ encoding mRNA and *piggyBac* plasmid. Genetic transformation was tested by amplification of the transgene via PCRs in male (drone) offspring (**b**). P is shown for fisher’s exact test for the significant difference only.

We used a previously published injection and rearing procedure^23^ to transform honeybee queens with transposon cassettes using the *piggyBac* (PB) transposon system. We first examined if we can directly target the germ cells and increase transformation rates in the offspring by injections in the posterior third of the egg. We suggested targeting this area because markers of the germ cells (the transcript of the *Am*-*vasa* and *Am*-*nos*) have been detected in cells that are arranged in a line from the 3^rd^ to the 6^th^ abdominal segment^27^. We obtained 9 queens out of 47 queens (19%; Fig. 1, Suppl. Fig. S2).

**Figure 2:**
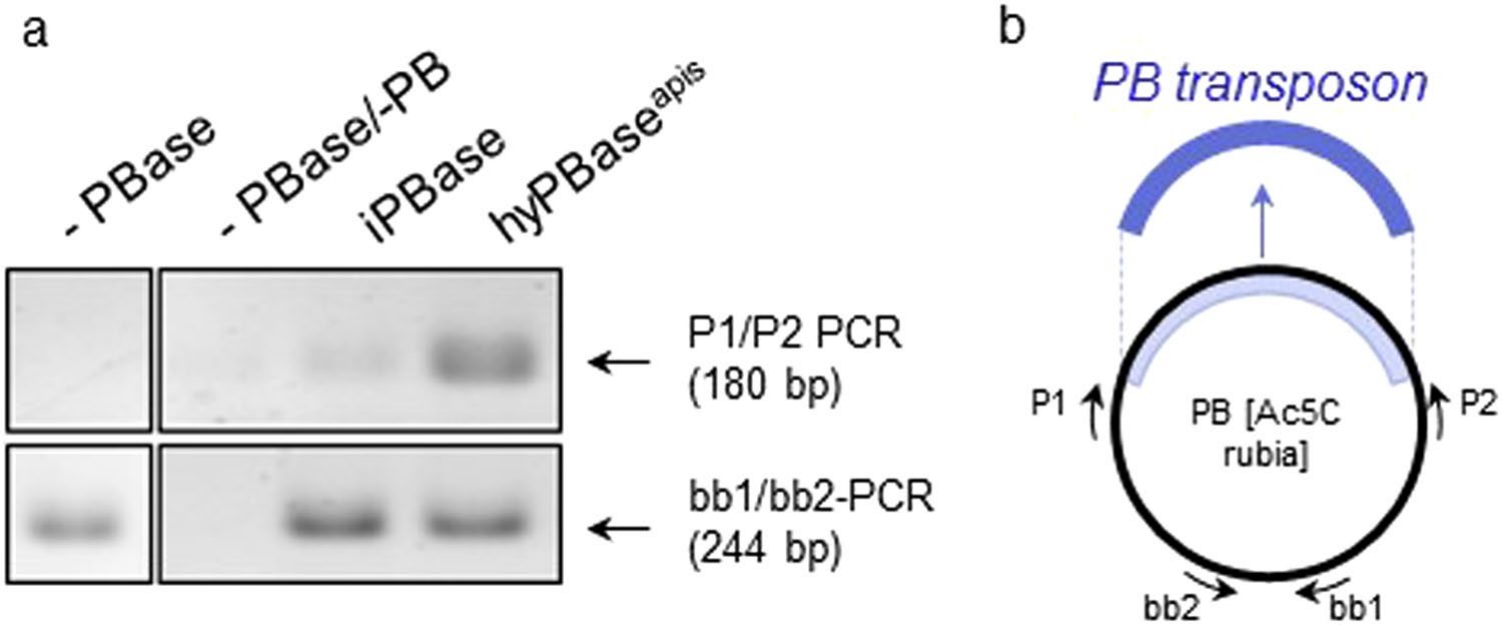
The excision of PB-transposon in response to the transposases iPBase and hyPBase^apis^ in *Sf*21 cells. (**a**) Semi-quantitative analysis of the amount of excised transposon using PCR. A total of 10^6^ cells were transfected with 1 µg of the PB[Ac5C rubia] plasmid^23^ and 1 µg of pIZ/V5-His PBase plasmid expressing either iPBase or hyPBase^apis^. We isolated plasmid DNA and detected PCR fragments only if the transposon was excised (P1/P2 PCR). The PCR reactions of the different treatments were semi-quantitatively standardized for the amount of transfected plasmid. To do so, we adjusted the template’s volumes in the PCR reactions so that they produced similar strong PCR products using the bb1 and bb2 primers. Fragments were resolved via gel electrophoresis and were visualized using ethidium bromide (figure was assembled from the same gel). (**b**) Schematic presentation of the targets of the two PCR reactions in the PB[Ac5C rubia] plasmid.

To possibly improve the transformation rate, we introduced the mutations of the hyperactive variant of the *piggyBac* transposase protein^24^ by synthesizing a gene that encoded the hyperactive mutations and adjusted the codon usage to that of the honeybee (hyPBase^apis^, Table 1). We compared the activity of hyPBase^apis^ to that of iPBase (insect *piggyBac* transposase from *Trichoplusia ni*, that is usually used in insects (reviewed in^28^)) using an excision assay in *Sf*21 cells, a permanent insect cell line which is routinely used in cell based assays^29^. We transfected cells with the *piggyBac* transposon provided by PB[Ac5C rubia] plasmid together with either the hyBPase^apis^ or the iPBase gene under the control of the OplE2 promoter (pIZ/V5-His vector). After 72 hours, we isolated plasmid DNA and semi-quantified the amount of excised transposon using PCRs (Fig. 2). In this semi-quantitative analysis (n = 3), we detected a strong PCR product (indicating the excision of the *piggyBac* transposon) when the hyPBase^apis^ gene was expressed and observed only a weak PCR product when the iPBase gene was expressed. These results provide a first indication that the hyPBase^apis^ gene can possibly provide a higher transposition activity in insect cells compared with the iPBase gene. Next, we examined the effect of hyPBase^apis^ on transformation rates in queens. We obtained 6 out of 27 queens (22%) (Fig. 1) with transgenic offspring.

**Table 1:** Modifications of the coding sequence of the iPBase gene to generate the hyPBase^apis^ gene.

Hyperactivation^24^	7 codons (I30V [GTA], S103P [CCA], G165S [TCT], M282V [GTG], S509G [GGA], N538K [AAA], N570S [TCT])
Codon-optimization	24 nucleotides (C81T, G264A, G315A, C378A, C390T, C409T, G480A, G696A, C699A, G786A, G831A, G843A, C1128A, C1158A, G1164A, C1200A, C1345T, C1350T, G1380A, G1392A, C1497A, C1587A, C1689A, G1701A)

To possibly further improve rates, we increased the amount of injected transposase mRNAs per embryo from 90 pg to 240 pg. This resulted in 31% queens with transgenic offspring (5 out of 16 queens, Fig. 1). Finally, we injected into the anterior region of the embryo (Fig. 1) expecting that this change would transform one of the first nuclei located in the anterior pole of the embryo^30^. This might be possible because our injections were performed 0 to 1.5 hours after egg deposition, while the first mitotic division occurs 3 to 4 hours after egg deposition. With the anterior injection site and the 240 pg of hyPBase^apis^ mRNA, we detected that 25 of 58 queens (43%) had transgenic offspring (Fig. 1). Injection with 240 ng of the new hyPBase^apis^ mRNAs at the anterior pole doubled the transformation rate as compared with injection with 90 pg of iPBase mRNAs in the posterior area (19 to 43%; d.f. = 1, fisher’s exact test P < 0.05).

To find further evidence of whether the change from posterior to anterior injection site may increase the chance of targeting the germ cells by transformation of the first nuclei, we compared how often we were able to detect the transgene in the ovaries of the queens generated from posterior and anterior injections with 240 pg of hyPBase^apis^ mRNA. We extracted genomic DNA from ovaries and amplified the transgene using PCRs (Suppl. Fig. S2a). We observed that 53% of the 49 queens from the anterior injection and only 30% of the 50 queens from the posterior injections (Suppl. Table S1) possessed ovaries with the transgene (Fisher’s exact test, P < 0.05). This result supports our hypothesis that anterior injections can more likely target the ovary tissue possibly by a transformation event in early embryogenesis.

**Figure 3:**
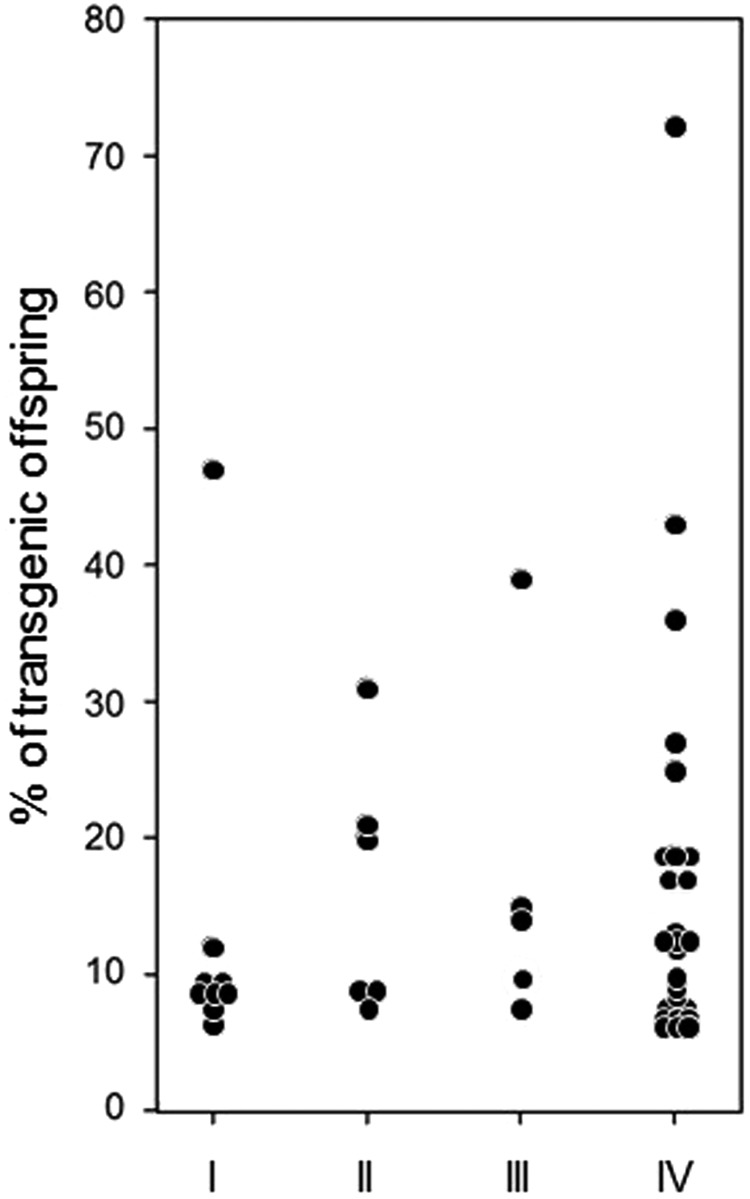
The relative proportion of offspring possessing the transgene for each of the transgenic detected queens. Because in some queens more than 16 individuals were analysed, we standardized for the expected number, if only 16 samples would have been examined. The conditions were as follows: (I) iPBase, 90 pg, posterior, tested queens N = 47. (II) hyPBase^apis^, 90 pg, posterior, tested queens N = 27. (III) hyPBase^apis^, 240 pg, posterior, tested queens N = 16. (IV) hyPBase^apis^, 240 pg, anterior, tested queens N = 58.

We next explored whether the improvements increase the percentage of offspring that possess the transgene. Although the proportion of transgenic offspring is in general quite large, we detected no evidence that the new procedures improved the proportion of transgenic offspring in the transformed queens (Fig. 3, cond. I–IV, Kruskal-Wallis-test, d.f. = 3, P = 0.7).

## Discussion

In this study, we demonstrated a twofold increase in transformation rates for 3.8- to 5.0-kb sized transposon cassettes using the *piggyBac* transposon system. In a previous study, we were able to produce transgenic queens with on average less than 10% of transgenic offspring (shown in^23^, therein presented in Table 2) suggesting that this low rate is not very supportive for further genetic experiments in honeybees. In this study, we employed a codon-adjusted hyperactive transposase gene, tripled the amount of transposase mRNA in the injections and changed the injection site from the posterior to the anterior site of the egg to target the first nuclei of the embryo. The implementation of all three procedures doubled the transformation rate from 19% to 43% of the queens with transgenic offspring. These improvements suggest that the analysis of gene functions using transposon cassettes (consisting of an endogenous promotor and gene as well as the flanking pBacL and R sequences) is feasible for an average bee facility.

Among the transgenic queens of the improved condition IV a single queen had more than 70% transgenic offspring. In combination with the observation that 26% of those transgenic queens produce more than 10% transgenic offspring, our results indicate that we can produce, with the improved transformation rates, queens with a large fraction of transgenic worker or male offspring in the first generation following the injected generation. This will offer the perspective to perform genetic experiments with the first generation and to do crossing experiments that require only a limited number of resources and honeybee colonies. Contrary to our assumption, it was not possible to transform the embryo at least before the first two nuclei divisions.

Our *Sf*21 cell experiments and transgenic ovary studies provide support that the hyperactive transposase expressed from a codon usage-adjusted gene and that the choice of the anterior injection site contributed to the overall increase in the transformation rate in honeybees. The expression of hyPBase^apis^ increased the number of plasmids in which the transposon was excised relative to the expression of the original iPBase. Further, the change from posterior to anterior injections increased the number of transgenic ovaries detected in the queens. We suggest that we have targeted with our anterior injections the first nuclei possibly located in that region^30^.

Similar improvements have been reported from experiments performed in other species, but not in this combination. Studies in mice demonstrated that the adjusted codon usage of the transposase gene from an insect to that of the mice increases the transposition efficiency up to 20-fold in mouse embryonic stem cells^25^, suggesting the importance of codon usage adjustments. The hyperactive transposase variant developed by Yusa *et al*. for mice increased the transformation rate by 10-fold^24^. Studies in the silk worm *Bombyx mori* reported that changes in the injection site and direction increased the transformation rates from 2.1% to 6.5%, suggesting the germ cells were better targeted^26^.

We propose that any of the steps - the use of hyPBase^apis^, the choice of injection site, and the amount of the injected transposase mRNA - or a combination of all three procedures can enhance transformation rates in other insect species, which have been reported to vary from 0.1% to 15% (reviewed in^28,31^). The further increased transformation rates would be especially rewarding for species in which the maintenance of females and the selection procedure in the offspring is as laborious as it is in honeybees.

## Materials and Methods

### Microinjection and rearing

Honeybee eggs (age: 1.5 hours after egg deposition) were injected and larvae reared to queens as previously described^23^. To induce egg laying, the treated queens were 9 d old when they were incubated with CO_2_ for 10 min on two successive days. One operator injected 30 pg of plasmid DNA in the years 2013–2015 and 10–15 pg in the year 2017 together with transposase mRNAs as previously described^23^. The plasmids used in this study (Suppl. Fig. S1) had a similar structure to those previously reported ([6xP3-rubia; Am-actin5c-egfp],^23^). The transposon cassettes (including pBacR and pBacL sequences) in this study ranged in size from 3.8 to 5.0 kb.

### Bee sources

The bees were feral colonies of the *A*. *mellifera carnica* strain.

### DNA Preparation, PCR, Nucleotide Analysis and mRNA Synthesis

Genomic DNA from honeybee drones was extracted from viable larvae at the 1–2 instar stage or from the dissected ovaries of the reared queens using the peqGOLD Tissue DNA Mini Kit (PEQLAB, Erlangen, Germany). Because in some queens more than 16 male (drone) offspring were analysed, it was necessary to standardize the percentages of transgenic offspring per queen to 16 for statistical analysis. Plasmid preparation, PCR reactions, use of restriction enzymes, sequencing and transposase mRNA synthesis were performed as previously described^23^.

### Cloning of the hyPBase^apis^ gene in pGEMT and pIZ/V5-His vector

The nucleotide sequence of hyPBase^apis^ (hyperactive and codon adjusted *piggyBac* transposase) was synthesized, and the DNA was cloned into the pUC57 vector (Centic Biotech, Weimar, Deutschland). We replaced the original transposase gene^32^ in pGEM-T iPBase^23^ with the hyPBase^apis^ sequence (nucleotide changes are shown in Table 1) using the ApaI/NcoI restriction sites. The resulting plasmid was named pGEM-T hyPB^apis^. Transposase mRNA was synthesized following the instructions of the mMESSAGE mMACHINE kit (Thermo Fisher Scientific, Dreieich, Germany). For the expression of the unmodified (iPBase) and the hyperactive (hyPBase^apis^) PB transposases under the control of the OplE2 promoter in *Sf*21 cells, we cloned the coding sequences into the pIZ/V5-His vector (Thermo Fisher Scientific, Dreieich, Germany) using the SacII/EcoRI restriction sites.

### Cell culture

*Sf*21 cells (Invitrogen, Carlsbad, US) were grown adherent (insect cell culture medium: Biochrom, Berlin, Germany) at 27 °C in six-well plates and maintained according to the manufacturer’s instructions. We transiently transfected 1 µg of *piggyBac* [Ac5C rubia] plasmid into 10^6^ cells using Roti Insectofect (Roth, Karlsruhe, Germany) in combination with 1 µg of either pIZ/V5-His iPBase or pIZ/V5-His hyPBase^apis^ transposase-expressing plasmid. After 72 hours, we isolated the plasmid DNA using the GeneJET Plasmid Miniprep Kit (Thermo Fisher Scientific, Dreieich, Germany).

### Excision assay

PCR oligonucleotide primers that amplified a 180-bp sequence if the transposon was excised (Fig. 2) were P1: CAGACAGCGTTGAGATATAC and P2: CAATGTGGTTTTTGTCAAACGAAG. The PCR oligonucleotide primers that amplified a fragment upstream of the Amp^R^ gene, which was not affected by the excision event, were bb1: CGACGTGTTGGCTAAAATTATTAAA and bb2: GCTGCAAGGCGATTAAGTTGGGTA.

## Electronic supplementary material

Supplementary Information

## References

[1] Seeley TD (1982). Adaptive Significance of the Age Polyethism Schedule in Honeybee Colonies. Behavioral Ecology and Sociobiology.

[2] Page RE, Erber J (2002). Levels of behavioral organization and the evolution of division of labor. Naturwissenschaften.

[3] Seeley, T. D. *The Wisdom of the Hive* (Harvard University Press, 1995).

[4] Robinson GE, Fernald RD, Clayton DF (2008). Genes and social behavior. Science..

[5] Zayed A, Robinson GE (2012). Understanding the relationship between brain gene expression and social behavior: lessons from the honey bee. Annu Rev Genet.

[6] Menzel R (2012). The honeybee as a model for understanding the basis of cognition. Nature reviews. Neuroscience.

[7] Menzel R, Giurfa M (2001). Cognitive architecture of a mini-brain: the honeybee. Trends Cogn Sci.

[8] von Frisch, K. *The Dance Language and Orientation of Bees*. (Harvard University Press, 1967).

[9] Riley JR, Greggers U, Smith AD, Reynolds DR, Menzel R (2005). The flight paths of honeybees recruited by the waggle dance. Nature.

[10] Robinson GE (1992). Regulation of division of labor in insect societies. Annu. Rev. Entomol..

[11] Beshers SN, Fewell JH (2001). Models of division of labor in social insects. Annual Review of Entomology.

[12] Whitfield CW, Cziko AM, Robinson GE (2003). Gene expression profiles in the brain predict behavior in individual honey bees. Science.

[13] Leoncini, I. *et al*. Regulation of behavioral maturation by a primer pheromone produced by adult worker honey bees. *Proc*. *Natl*. *Acad*. *Sci*. *USA* (2004). 10.1073/pnas.0407652101 PMC536028 15572455

[14] Bonabeau E, Theraulaz G, Deneubourg JL, Aron S, Camazine S (1997). Self-organization in social insects. Trends in ecology & evolution.

[15] Theraulaz G, Bonabeau E, Deneubourg JL (1998). Response threshold reinforcement and division of labour in insect societies. P Roy Soc B-Biol Sci.

[16] Vleurinck C, Raub S, Sturgill D, Oliver B, Beye M (2016). Linking Genes and Brain Development of Honeybee Workers: A Whole-Transcriptome Approach. PloS one.

[17] Beye M, Hasselmann M, Fondrk MK, Page RE, Omholt SW (2003). The gene *csd* is the primary signal for sexual development in the honeybee and encodes an SR-type protein. Cell.

[18] Beye M (2013). Gradual molecular evolution of a sex determination switch through incomplete penetrance of femaleness. Current biology: CB.

[19] Haydak Mykola H. (1970). Honey Bee Nutrition. Annual Review of Entomology.

[20] Kamakura M (2011). Royalactin induces queen differentiation in honeybees. Nature..

[21] Buttstedt A, Ihling CH, Pietzsch M, Moritz RF (2016). Royalactin is not a royal making of a queen. Nature.

[22] Kucharski R, Maleszka J, Foret S, Maleszka R (2008). Nutritional control of reproductive status in honeybees via DNA methylation. Science..

[23] Schulte C, Theilenberg E, Muller-Borg M, Gempe T, Beye M (2014). Highly efficient integration and expression of piggyBac-derived cassettes in the honeybee (Apis mellifera). Proc Natl Acad Sci USA.

[24] Yusa K, Zhou L, Li MA, Bradley A, Craig NL (2011). A hyperactive piggyBac transposase for mammalian applications. Proc Natl Acad Sci USA.

[25] Cadinanos J, Bradley A (2007). Generation of an inducible and optimized piggyBac transposon system. Nucleic acids research.

[26] Tamura T, Kuwabara N, Uchino K, Kobayashi I, Kanda T (2007). An Improved DNA Injection Method for Silkworm Eggs Drastically Increases the Efficiency of Producing Transgenic Silkworms. JIBS.

[27] Dearden PK (2006). Germ cell development in the Honeybee (Apis mellifera); vasa and nanos expression. BMC Dev Biol.

[28] Gregory M, Alphey L, Morrison NI, Shimeld SM (2016). Insect transformation with piggyBac: getting the number of injections just right. Insect molecular biology.

[29] Genersch, E. *et al*. Standard methods for cell cultures in Apis mellifera research. *J Apicult Res* **52**, doi:Artn 52.1.0210.3896/Ibra.1.52.1.02 (2013).

[30] Schnetter M (1934). Morphologische Untersuchungen über das Differenzierungszentrum in der Embryonalentwicklung der Honigbiene. Zeitschrift für Morphologie und Ökologie der Tiere.

[31] Morrison NI (2010). Genetic Improvements to the Sterile Insect Technique for Agricultural Pests. AsPac J. Mol. Biol. Biotechnol.

[32] Berghammer AJ, Klingler M, Wimmer EA (1999). A universal marker for transgenic insects. Nature.

