# Supplementary material for: Improving genetic transformation rates in honeybees
Source: Sci Rep. 2018 Nov 8;8:16534. doi: 10.1038/s41598-018-34724-w (PMC6224437; doi:10.1038/s41598-018-34724-w)
Supplement: Supplementary file 1 — Supplementary Information [file 41598_2018_34724_MOESM1_ESM.doc]

**Supplementary Information**

**Improving genetic transformation rates in honeybees**

M. Otte^1*^, O. Netschitailo^1^, O. Kaftanoglu^2^, Y. Wang^2^, R.E. Page Jr.^2,3^, M. Beye^1^

^1^ Evolutionary Genetics, Heinrich Heine University Düsseldorf, Germany

^2^ School of Life Sciences, Arizona State University Tempe, United States

^3^ Department of Entomology and Nematology, University of California Davis, United States

*corresponding author: M. Otte: [otte@hhu.de](mailto:otte@hhu.de)


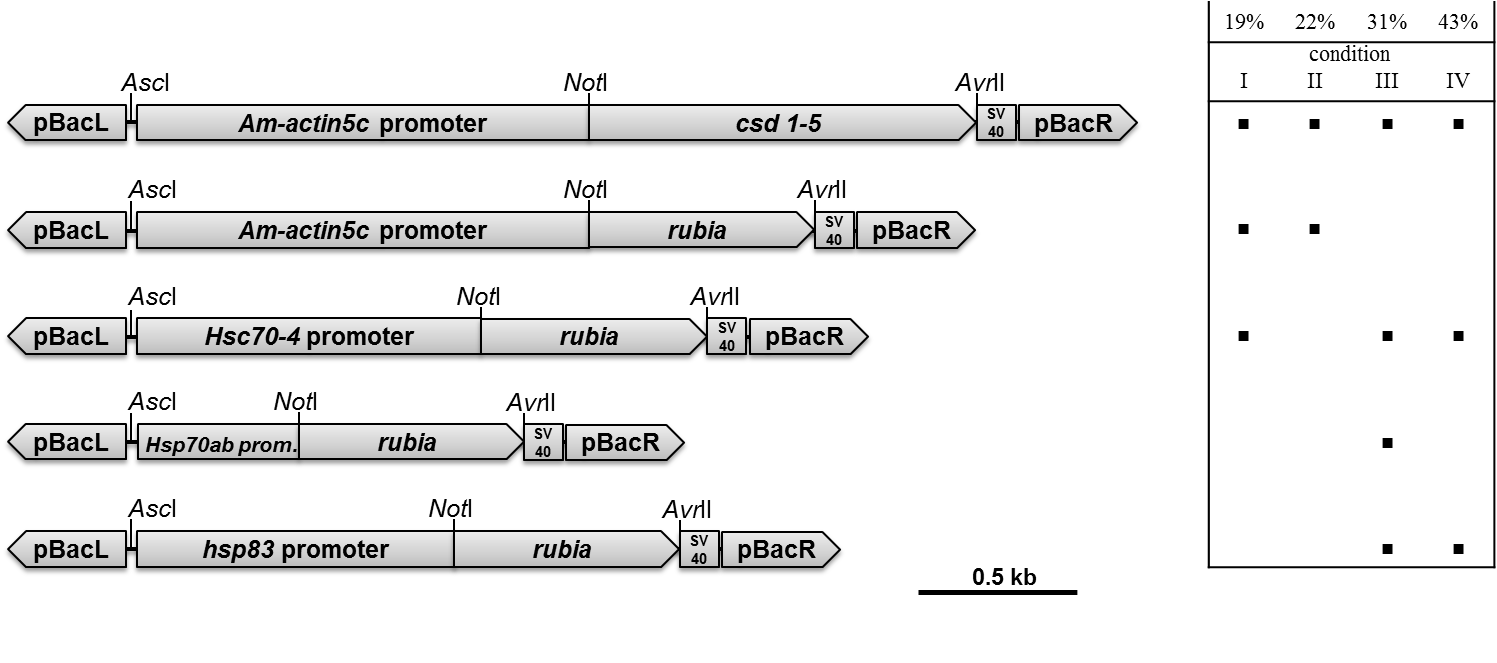


**Supplementary Figure S1:** Structure of the transposon cassettes with the piggyBac transposon elements used in the different procedures. The boxes indicate the structural elements. BacR, BacL: inverted terminal repeats of the piggyBac transposon, including parts of the transposase coding sequence; *Am-actin5c* promoter: a 1420-bp sequence upstream of the translation start site of the honeybee *actin5c* gene; *Am-hsc70-4*: a 1091-bp sequence upstream of the translation start site of a potential honeybee *hsc70-4* gene; *Am-hs70ab*: a 481-bp sequence upstream of the translation start site of a potential honeybee *hsp70ab* gene; *Am-hsp83*: a 1010-bp sequence upstream of the translation start site of a potential honeybee *hsp83* gene; *Rubia* and *egfp*: reporter genes encoding red or green fluorescent proteins, respectively; *csd* 1-5: five sequences from the honeybee *csd* gene; SV40: the *SV40* polyadenylation site. The letters above the figure denote the restriction sites that were used for cloning. The scale indicates the size in kilobases (kb).

The procedure (conditions) were the following: I: iPBase, 90 pg, posterior; II: hyPBase^apis^, 90 pg, posterior; III: hyPBase^apis^, 240 pg, posterior; IV: hyPBase^apis^, 240 pg, anterior. Percentages above the condition number indicate the amount of transgenic queens with transgenic offspring.

**Supplementary Table S1:** Number of queens (N) possessing transgenic ovaries, either injected at the anterior or posterior site with 240 pg of hyPBase^apis^ mRNA.

|  |  | Queens with transgenic ovaries | |
| --- | --- | --- | --- |
| Injection site |  | Yes | No |
| Posterior | N=50 | 30%  (15) | 70%  (35) |
| Anterior | N=49 | 53%  (26) | 47%  (23) |


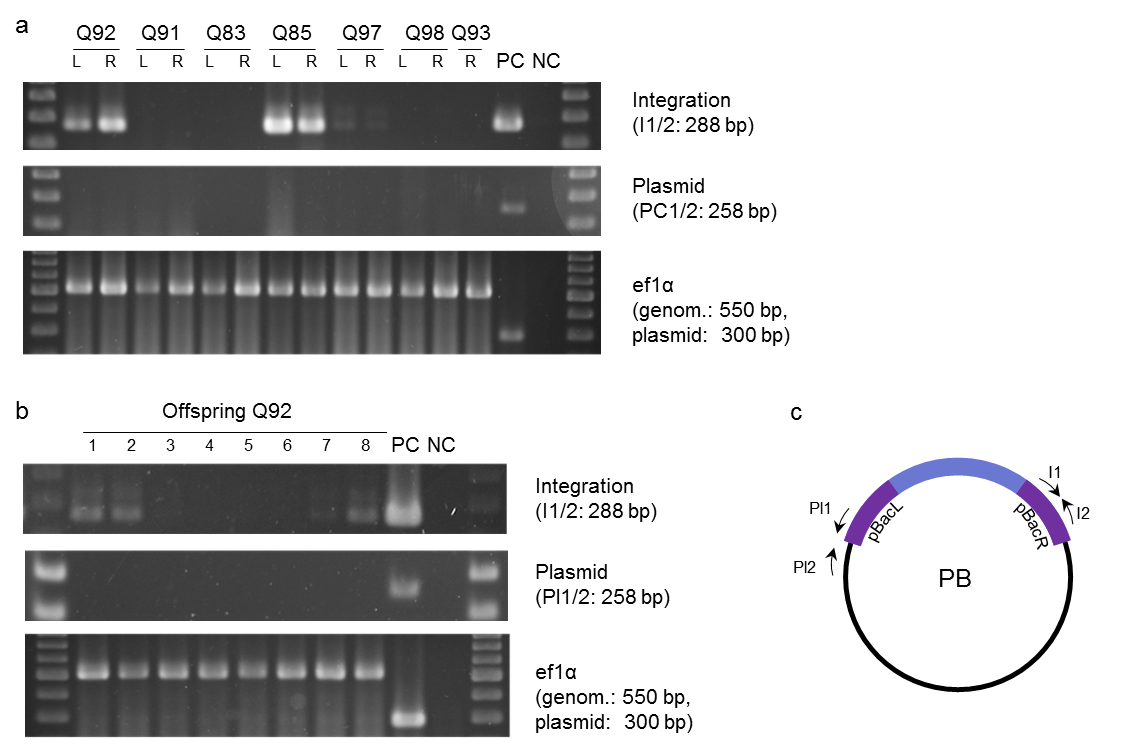


**Supplementary Figure S2. Stable genomic integration and transmission of the transposon cassette.** We studied PCRs from genomic DNA of ovary tissue (a) and single offspring (b) that were in three separate reactions: one amplifies specifically the sequence of pBacR, showing the presence of the transposon (upper picture in (a) and (b)); a second PCR amplifies the junction of the plasmid and the transposon, indicating the presence of the piggyBac plasmid in the offspring (middle picture in (a) and (b)). The next PCR amplifies two exons and one intron of the *elongation factor*1α (*ef*1α) gene (lower picture in (a) and (b)) that served as control for DNA and PCR quality. Fragments were separated by agarose gel electrophoresis. PC denotes the control reactions using the injected plasmid DNA as template, NC denotes the control reaction with no DNA.

(c) Schematic presentation of binding sites of the oligonucleotide primers at PB plasmid. The PCR primers were: Pl1:GCTGCAAGGCGATTAAGTTGGGTA; Pl2:CGACGTGTTGGCTAAAATTATTAAA; I1:CCACACCTCCCCCTGAACCTGAAAC; I2:GAGGTAAGAATAAACATTGTTGGTC. Primer sequences for *ef*1α gene are: ef1:CGTTCGTACCGATCTCCGGATG; ef2:GCTGCTGGAGCGAATGTTAC.
